# Factors Affecting the Sustainment, Sustainability, and Spread of Practice Changes in Canadian Long-Term Care Homes

**DOI:** 10.1093/geroni/igab046.1593

**Published:** 2021-12-17

**Authors:** Lauren MacEachern, Yuting Song, Liane Ginsburg, Malcolm Doupe, Adrian Wagg, Jude Spiers, Whitney Berta

**Affiliations:** 1 University of Toronto, Toronto, Ontario, Canada; 2 University of Alberta, Edmonton, Alberta, Canada; 3 York University, York University, Alberta, Canada; 4 University of Manitoba, Winnipeg, Manitoba, Canada; 5 University of Toronto, University of Toronto, Ontario, Canada

## Abstract

Our understanding of the post-implementation sustainment, sustainability, and spread (SSS) of complex quality improvement interventions is limited. We explored factors that influenced the SSS of a care aide-led quality improvement initiative (Safer Care for Older Persons (in residential) Environments [SCOPE]) implemented in 6 Manitoba long-term care homes two years after the conclusion of SCOPE in 2017. We analyzed small group interview data collected from all unit- and facility-level managers who participated in SCOPE and were still working in these facilities. We asked about SCOPE implementation, post-SCOPE quality improvement activities, factors that influenced them, and about inter-unit spread of SCOPE following the project’s conclusion. The interviews were audio-recorded, transcribed verbatim, de-identified, and analyzed using thematic analysis. Five of the 6 facilities reported sustained SCOPE quality improvement activities, tools, and facilitative structures. In the same 5 facilities, SCOPE benefits (e.g., increases in care aide empowerment and self-efficacy, manager belief in care aide capacity) continued post-implementation. Spread beyond the original SCOPE units had occurred in 3 facilities. Factors that influenced the SSS of SCOPE were related to the team (e.g., care aides' quality improvement capacity), to the unit and facility (e.g., culture of innovation and change), and to the long-term care system (e.g., competing imperatives). Some factors influencing SSS differ from factors known to influence implementation. The identified factors affecting SSS highlight the influence of social dynamics (i.e., interactions, communication, relationships) among staff on SSS. Further research is warranted to explore interactions among these influencing factors and how they lead to SSS.

